# Development of rapid and cost-effective top-loading device for the detection of anti-SARS-CoV-2 IgG/IgM antibodies

**DOI:** 10.1038/s41598-021-94444-6

**Published:** 2021-07-21

**Authors:** Essam H. Ibrahim, Hamed A. Ghramh, Mona Kilany

**Affiliations:** 1 Biology Department, Faculty of Science, King Khalid University, P.O. Box 9004, Abha, 61413 Saudi Arabia; 2 Research Center for Advanced Materials Science (RCAMS), King Khalid University, P.O. Box 9004, Abha, 61413 Saudi Arabia; 3 Blood Products Quality Control and Research Department, National Organization for Research and Control of Biologicals, Cairo, Egypt; 4 Unit of Bee Research and Honey Production, Faculty of Science, King Khalid University, P.O. Box 9004, Abha, 61413 Saudi Arabia; 5 Department of Microbiology, National Organization for Drug Control and Research (NODCAR), Cairo, Egypt

**Keywords:** Biological techniques, Biological models, Immunological techniques

## Abstract

Infection with SARS-CoV-2, the Betacoronavirus, caused a pandemic that affected the globe negatively. The gold method, RT-PCR, can detect SARS-CoV-2 but it is time-consuming and needs sophisticated equipment and professional personnel. On the other hand, rapid tests offer fast results and can detect anti-SARS-CoV-2 antibodies (Abs). The aim of this study is to develop a new rapid and cost-effective method for the detection of anti-SARS-CoV-2 IgG/IgM Abs. A new top-loading detection device was developed and composed of a small piece of plastic (25 × 25 × 0.5 mm) with an opening in the center, a piece of nitrocellulose (NC) membrane enough to block the opening from one side and adhesive tape to affix the NC to the plastic piece. The NC is blotted with anti-human IgG/IgM and rabbit serum. The device was evaluated against a commercially available IgG/IgM ELISA detection kit using normal, Covid-19-positive, HCV, HBV, and Cytomegalovirus-positive sera. Outcomes demonstrated simplicity, reproducibility, and accuracy of the new device and results can be obtained in less than 5 min. We anticipate our developed assay method to be used widely in point of care before deciding on the use of expensive nucleic acid assays.

## Introduction

The immune system is an effective guard against invasions of pathogens on the human body and it has many strategies to perform its tasks. The preexisting defense mechanism, the innate immunity, is the first defense to prevent body infection by microbes. It can be rapidly activated by invading microbes before going ahead to the adaptive immunity^1^. Many pathogenic microbes can escape innate immunity using strategies that are fateful for the microbes virulence^2^. Adaptive immunity is potent and specialized and able to eliminate microbes escaped from innate immunity^3^. The effector mechanisms of innate immunity are often used by adaptive immunity to eliminate microbes. In humoral immunity, B cells synthesize antibodies to eliminate microbes through the complement system and phagocytes, the two effector mechanisms of innate immunity^4^. Cell-mediated immunity, mediated by T cells, is the effector function against pathogens that get by inside cells^5^.

The severe acute respiratory syndrome coronavirus 2 (SARS-CoV-2), the RNA virus appeared during 2019, is well-described and analyzed^6–8^. SARS-CoV-2 enters the host cell after the binding of the spike (S) protein which presents on the surface of SARS-CoV-2 to the angiotensin-converting enzyme 2 (ACE2) receptor presents on the host cell surface enabling the entrance and propagation of the virus within the cell with a limited innate immune response. When the virus proliferates and arrives at the respiratory tract, it faces a stronger innate immune response turning the illness to being clinically manifested^9,10^.

The immunoglobulin IgA and IgM seroconversion peaks, as a result of SARS-CoV-2 exposure, after approximately 4–6 days, while it peaks after at least 10 days for IgG^11,12^. A study by Zhao et al.^12^ showed that the average days for seroconversion post symptom beginning for total antibodies, IgM and IgG were 11, 12 and 14 days respectively. Antibodies recognition in mild cases can take a longer time (a month or more) and in a few cases IgM and IgG are not recognized at all. Cellular reactions against the S protein have been portrayed and found to associate with IgG and IgA antibody titers^13,14^. Total lymphocytes (CD4^+^/CD8^+^ cells, B-lymphocytes and NK cells) showed a significant correlation with inflammatory status in SARS-CoV-2 infection, with special regards to CD8^+^ T cells and CD4^+^/CD8^+^ ratio^15^. Decreased absolute numbers of T cells (CD4^+^/CD8^+^) were clear in mild cases and severe cases but exaggerated in the severe cases. The CD4^+^ T cells IFN-γ production was shown to be decreased in severe cases than in moderate cases^16^.

The rapid genomic sequence analysis of SARS-CoV-2 facilitated the design of reverse-transcription polymerase chain reaction (RT-PCR) methods and antibody-based diagnostics^17^. RT-PCR methods can detect the presence SARS-CoV-2 in upper respiratory tract specimens. The majority of the antibody-based methods depend on recombinant S protein. Some use the nucleoprotein (N), but taking into account that N is more conserved among coronaviruses and the possibility of cross-reactivity. Several laboratory assays are validated, and several antibody data were accrued^18–22^. There are several formats of antibody tests, including rapid lateral flow devices, enzyme-immunoassay techniques, and virus neutralization assays. Antibody diagnostic tests are useful in discovering the asymptomatic and/or recovered persons^23^.

In respect of its low-cost, fast, sensitive, low limit of detection and specific diagnostic power, the lateral flow immunoassay (LFA) technique became the popular diagnostic method in recent years^24^. This technique can be helpful in the detection of the SARS-CoV-2 infection, offering a rapid diagnosis of suspected patients. Thousands of lateral flow assay kits have been developed since the emergence of SARS-CoV-2^25^. Different formats are adopted in LFA but all have a basic structure. LFA contains four main components; sample application pad, conjugate pad, nitrocellulose membrane and adsorbent pad^26^. Sample application pad is made of cellulose or glass fiber. To that pad, the sample is applied to start the assay. Conjugate pad is the place where labeled biomolecules are dispensed. Liquid sample diffusing from the sample pad reacts with materials found in the conjugate pad, releases labeled biomolecules and moves them toward the nitrocellulose membrane. Nitrocellulose membrane has test and control lines. It is ideal for providing good support and binding to capture probes (antibodies or antigen). Adsorbent pad is found at the end of the strip and works as a sink. It works to keep a continuous flow of the liquid over the membrane and prevents back flow of the sample.

Taking into consideration the studies completed on SARS and MERS, the immune responses of exposed individuals would be focused on antibody responses to the S protein ^27–29^. So, in this study, we aimed to design a new format of colloidal gold-based immune-diagnosis using the S protein and a top-loading method.

## Results

### Characterization of synthesized AuNPs

**Figure 1:**
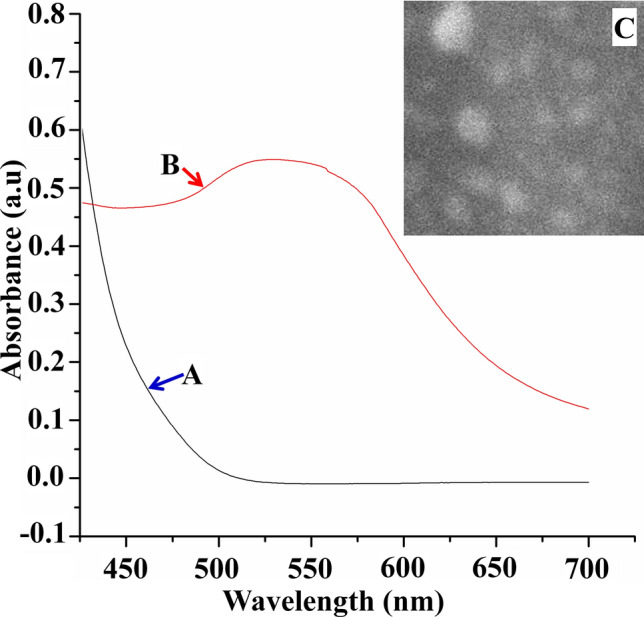
Gold nanoparticle synthesis. (**A**) gold chloride solution; (**B**) after the formation of AuNPs; (**C**) SEM image showing well-dispersed colloidal AuNPs.

Gold nanoparticles (AuNPs) were shown to be synthesized at a peak of 520–570 nm (Fig. 1A,B) with spherical shape (Fig. 1C).

**Figure 2:**
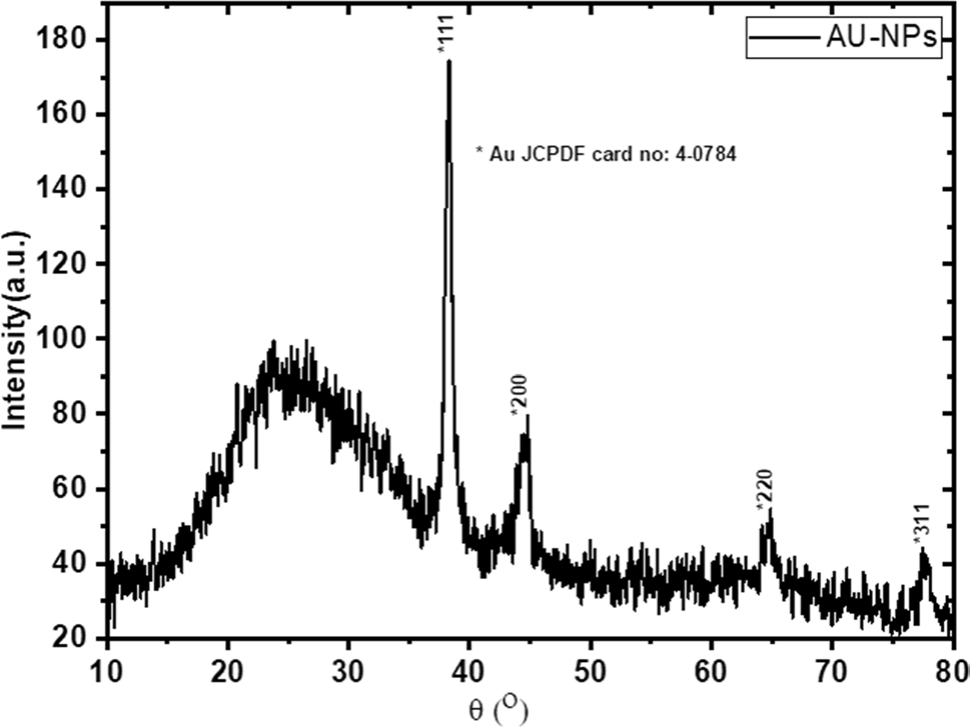
XRD analysis of AuNPs.

The powder X-ray diffraction (PXRD) pattern (Fig. 2) displayed some diffraction peaks at ca. 38.29°, 44.52°, 64.69° and 77.45°, which are well-indexed to the (111), (200), (220) and (311) facets of the typical fcc phase of gold (JCPDS no. 04–0784), respectively ^30^. Other diffraction peaks may be attributed to the organic materials (citrate) during preparation. The average grain size was calculated to be 17.11 nm (Table 1)^31^.

**Table 1:** The average particle size (D) calculated from XRD, d-spacing of different diffracting plans.

Peak index	2Theeta	FWHM(β)	D (nm)	d (A^0^)
(111)	**38.290**	**0.642**	13.104	**2.354**
(200)	**44.520**	**0.88**0	9.758	**2.033**
(220)	**64.695**	**0.41**0	22.942	**1.440**
(311)	**77.455**	**0.45**0	22.634	**1.231**
The average particle size ( XRD )			17.110	

### Optimization of protein concentration and pH value

**Figure 3:**
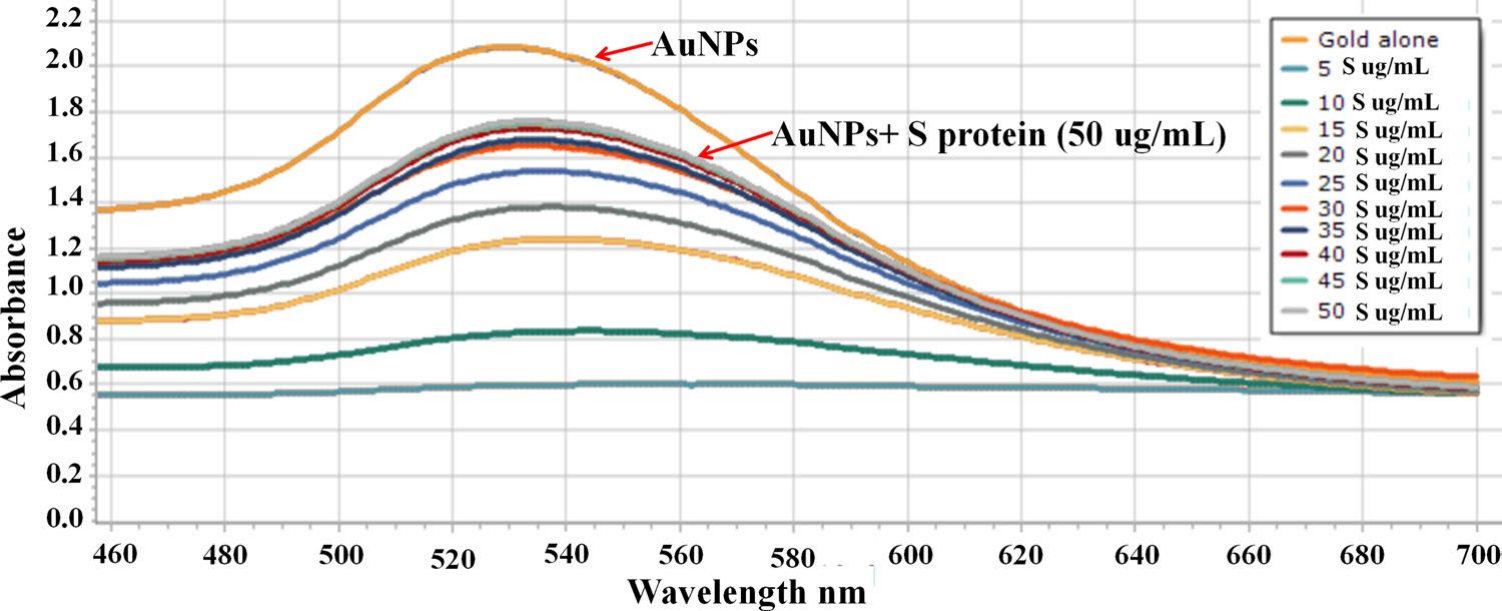
Spectral analysis of different protein concentrations that prevented AuNPs precipitation.

The concentration of the S protein that is capable of preventing the clumping of AuNPs in the presence of 10% NaCl was determined. Protein concentration of 10 µg/mL started to protect AuNPs from precipitation by 10% NaCl. Protein protection continued to increase until the concentration of 50 µg/mL (Fig. 3).

Optimal pH for S:AuNPs conjugation was tested in the pH range of 4.2–10.6 by using 40 µg S protein/mL AuNPs. Optimal pH for S:AuNPs conjugation was shown to be stable at all tested pH values (4.2–10.6) (Supplementary Fig. S1 online). We continued using the pH 8.2 for further work.

### S-colloidal gold conjugate probe

The S protein was mixed with colloidal AuNPs to get S-colloidal gold conjugate. The final S-gold conjugate was tested using SEM analysis and the SEM image revealed aggregates of spherical coated AuNPs (Supplementary Fig. S2 online).

### Sensitivity of TLTD

**Figure 4:**
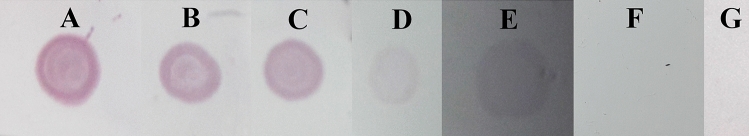
Titration of COVID-19 positive and negative sera. Where COVID-19 positives serum was diluted to A: 1:2; B: 1:4, C: 1:8; D: 1:16and E: 1:32. Negative serum (F) was diluted at 1:2. G: Blank (no serum).

Sera were serially diluted in wash buffer up to 1:128 and its reactivity against S protein was tested. Results showed that antibodies were detectable up to the dilution 1:16 in some sera, but it was good for all sera at the dilution 1:4 (Fig. 4).

### Testing of samples for IgG and IgM antibodies

All samples (COVID-19 PCR positive and negative) were tested for anti-SARS-CoV-2 IgG and IgM antibodies using the commercially available ELISA kit. The results of ELISA greatly matched those obtained by the top-loading test device (TLTD), but with the discrepancy in the intensity of the reaction in some sera. All control samples showed no IgG and IgM reactive antibodies against SARS-CoV-2.

**Figure 5:**
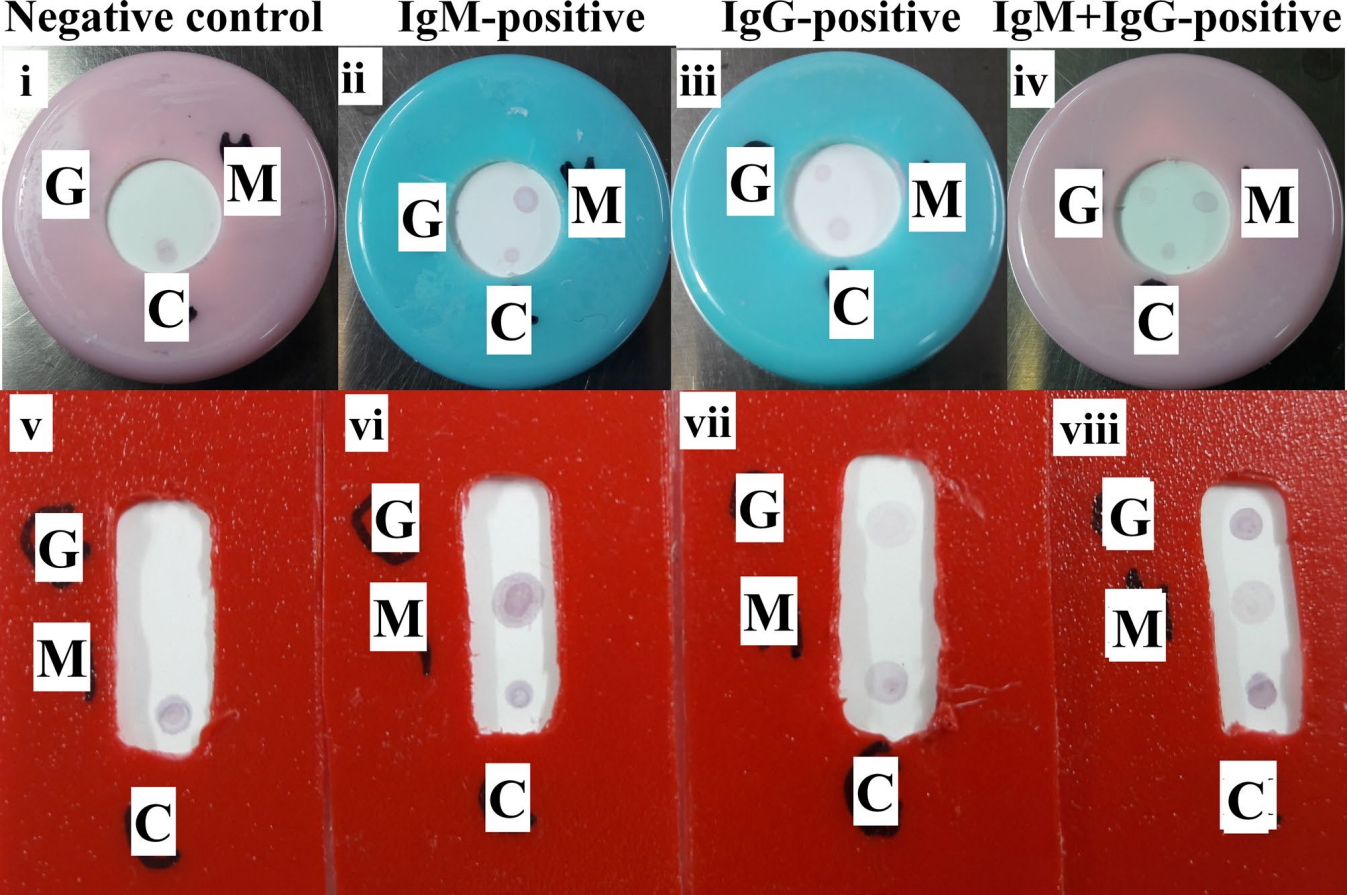
Testing of COVID-19 PCR positive sera using TLTD format 1 (i–iv) and format 4 (v–viii). Where G: expected positive area for IgG against S protein; M: serum positive for IgM against S protein and C: positive control. Column Negative control: normal serum; Column IgM-positive: anti-SARS-CoV-2 IgM Abs positive serum; Column IgG-positive: anti-SARS-CoV-2 IgG Abs positive serum and Column IgM + IgG-positive: anti-SARS-CoV-2 IgM and IgG positive serum. All sera at the dilution 1:4.

Patient sera showed different reactivity regarding IgG and IgM depending on the case, but most of them were IgM reactive, either alone or showing faint dot at IgG place. Many samples were negative for both IgM and IgG and only 2 samples were positive IgG alone (Fig. 5 and Supplementary Fig. S3 online).

### Specificity of TLTD

To determine specificity of the TLTD for COVID-19 detection, normal and HCV, HBV, HIV, and cytomegalovirus-infected sera were tested using TLTD. No positive reaction was seen using these sera (Supplementary Fig. S4 online) indicating the specificity of TLTD for Covid-19. Also, the TLTD was tested against normal (syphilis, malaria, HCV, HBV, HIV, HTLV and cytomegalovirus-free) sera and was found to be non-reactive to any of the tested sera (Supplementary Fig. S5 online).

### Reproducibility and stability of VID

Results indicated reproducibility and stability of the TLTD formats 2–4. Format 1 showed non-reproducible results as dots either appeared with different intensities or did not appeared at all.

## Discussion

SARS-CoV-2, the positive RNA virus, caused global health and economic disturbances. A new genetic mutation appeared in the Great Britain to begin a new episode of the pandemic. Accurate and rapid diagnosis is required in any disease including Covid-19. At the onset of COVID-19 symptoms, sera from infected persons contained detectable anti-SARSA-CoV-2 Abs ^32^. qPCR, the method which depends on the detection of the viral RNA, is used as the gold-standard method for the diagnosis of SARS-CoV-2 infection ^33^. Notwithstanding, numerous labs and medical clinics do not have the facilities and expertise needed to run the qPCR. Point-of-care (POC) needs rapid and simple diagnostic tests that are designed to shorten the sample-to-result timeline, enabling fast diagnosis and right treatment ^34^. Hence, there is a growing utilization of rapid tests for the diagnosis of Covid-19, giving the advantage to quickly affirm the infection. Lateral flow assays are the most widely used form in POC because of its low-cost and ease of use. In addition to rapid assay tests, ELISA-based assays are useful in detecting the antibodies directed against the SARS-CoV-2.

Here, we tried to create a new method for the detection of coronavirus infection at antibody level. We developed a new device that uses fewer materials to build up and does not need highly professional personnel to perform the test at any POC location. A lateral flow device is composed of sample pad, conjugate pad, NC membrane and absorbing filter paper affixed together on a plastic backing and packed into a plastic device ^35^. TLTD is composed only of NC membrane affixed to adhesive tape and a piece of square plastic with an opening. This demonstrates how TLTD is simple, economic and easy to assemble.

We performed the test using four formats. Format one had been previously used to detect other diseases such as Leptospirosis ^36^, food allergen ^37^, HIV-1 ^38^, etc.

Before evaluating the formats described in this study, all samples were tested for COVID-19 IgG and IgM using a commercially available ELISA kit. The plates of the kit are coated with the S protein. This matches our created TLTD as both will measure only IgG and IgM directed against the S protein.

Format 1 showed non-reproducible results as dots either appeared with different intensities or did not appear at all. This may be due to the manual assembly of the device, no enough time for antibodies interactions or this method is not good enough. So we decided to use another formats.

Format 3 and 4 uses fewer amounts of liquids (serum and conjugate) as compared to format 2 as the thickness of the plastic used to build the body of formats 3 and 4 is less than that of format 2. The antibody detection results of formats 2–4 were comparable to that obtained by ELSA. Most of the qPCR-positive samples were IgM-positive, some were IgG/IgM-negative, little IgG-positive and many IgG/IgM-positive. The positive sera for anti-S IgM antibodies may be due to variables such as most of the patients are in early phase of immune response where the IgM still dominant and the IgG antibodies started to appear or have still not appeared.

All HCV, HBV, HIV, and cytomegalovirus-infected sera gave negative results when tested using TLTD indicating the specificity of TLTD for Covid-19. Also, when testing the TLTD against syphilis, malaria, HCV, HBV, HIV, HTLV and cytomegalovirus-free sera, the TLTD was found to be non-reactive to any of the tested sera indicating that the device in not reactive with any component of SARS-CoV-2-free human sera. So, we can conclude that TLTD does not cross react with other viruses and is negative for the uninfected sera.

The purpose of making the TLTD in these formats is to use it in more remote areas or POC before deciding to choose qPCR assay. The transport and storage without the use of a cold chain is a great advantage of any product. Here, we tested the stability of TLTD after incubating it at different temperatures. The results reflected the ability of TLTD to tolerate high temperature (37 $^\circ$C) for a long time (30 days), giving an advantage to the newly designed device.

Results indicated reproducibility and stability of the TLTD formats 2–4 but format 1 was non-reproducible.

## Conclusion

A new design of rapid antibody detection device was developed. The new device is simple, accurate, reproducible, and cost-effective. It is very suitable for the detection of IgG and IgM arisen in SARS-CoV-2-infected persons in point of care and remote areas. The device is easy to use and does not need highly professional personnel to perform. It overcomes the need for sophisticated equipment like the PCR system for the diagnosis in POC and in remote areas and can be completed within minutes.

## Methods

### Preparation of colloidal gold nanoparticles

Nano gold was synthesized via the tetrachloroauric(III) acid (HAuCl_4_.3H_2_O, Honeywell Fluka) reduction as per Frens ^39^ procedures. Gold chloride (200 mL, 0.01% w/v, Sigma-Aldrich) solution was boiled (2 min), 4 mL of 1% (w/v) of tri-sodium citrate (Sigma-Aldrich) solution was then added rapidly and continued by heating until a wine-red color developed. The mixture was left to cool down to reach ambient temperature before storing at 4 °C.

### Characterization of colloidal AuNPs

Gold nanoparticles (AuNPs) formation was monitored using UV/Vis spectrophotometry (Genesys 10uv Scanning, Thermo Scientific). Morphology of AuNPs was detected using a scanning electron microscope (SEM, JSM-7500 F; JOEL-Japan) at 80 kV. The powder X-ray diffraction (PXRD) measurement was carried out by X-ray diffractometer (Shimadzu LabX-XRD-6000 diffractometer) run at 40 kV and 35 mA and a spectrum was recorded by CuKα radiation with a wavelength of 1.5406 Å in the 2θ range of 10°–80° with Shimadzu software with the pdf-2 library for the analysis of XRD data. The average crystallite size (D), was estimated by Debye- Scherer's Eq. ^40^:

1 $$ D = \frac{k\lambda }{{\beta \cos \theta }} $$

where the shape factor k is constant, equals 0.9 (k is dimensionless), X-ray's beam wavelength λ in nm, β is known as the full width at half maximum (FWHM) of the peak in radian and the Braggs’ diffraction angle is θ in degree.

### Optimization of pH and protein concentration for the AuNPs conjugation

The concentration of the S protein (Cusabio Technology LLC., China) that is capable of preventing the clumping of AuNPs in the presence of 10% NaCl (Sigma-Aldrich) was determined and this concentration was selected for further use. First the pH of the colloidal gold was adjusted to 8.2 with 0.1 M K_2_CO_3_ (Sigma-Aldrich). Various amounts of S protein (0, 5, 10, 15, 20, 25, 30, 35, 40, 45 and 50 µg) were individually mixed with 1 mL AuNPs and incubated for 10 min with shaking at room temperature. After that, 10% NaCl (100 µL/tube) was added, continued shaking for 10 min and then left standing for 2 h and each tube was scanned spectrophotometrically (460–700 nm). The amount of protein showed maximum absorbance at about 520 nm was considered as the minimum quantity of S protein required to prevent the precipitation of colloidal gold.

Optimal pH for S:AuNPs conjugation was tested in the pH range of 4.2–10.6 by using 40 µg S protein/mL AuNPs and testing the proper conjugation as described above.

### Preparations of S-AuNPs conjugate

The S protein (at a final concentration of 40 µg/mL) was mixed with 10 mL of AuNPs (pH 8.2) and gently shaken for 30 min. The conjugation process was stabilized via the addition of 2.5 mL of 10% BSA (Sigma-Aldrich) while gently shaking for 30 min. After that, 250 µL of 5% PEG20,000 (Sigma-Aldrich) solution (with a final concentration of 0.1%) was added to the mixture and left at room temperature while gently stirring for 16 min. The S-AuNPs conjugate was centrifuged at 14,000 rpm (10 min/4 °C), the supernatant was discarded and the pellet was resuspended in 1 mL storage buffer (phosphate-buffered saline (PBS, 137 mM NaCl, 2.7 mM KCl, 10 mM Na_2_HPO_4_, and 1.8 mM KH_2_PO_4_, pH 7.4) containing 5% sucrose, 2% bovine serum albumin (BSA), 0.5% Tween-20, and 0.03% NaN_3_). The final S-gold conjugate was stored at 4 $^\circ$ C for further study. The conjugate was tested using SEM analysis.

### Construction of the top-loading test device

We prepared four formats of top-loading test device (TLTD) for the detection of human IgG and IgM antibodies (Abs) against S protein of SARS-CoV-2 as described.

**Figure 6:**
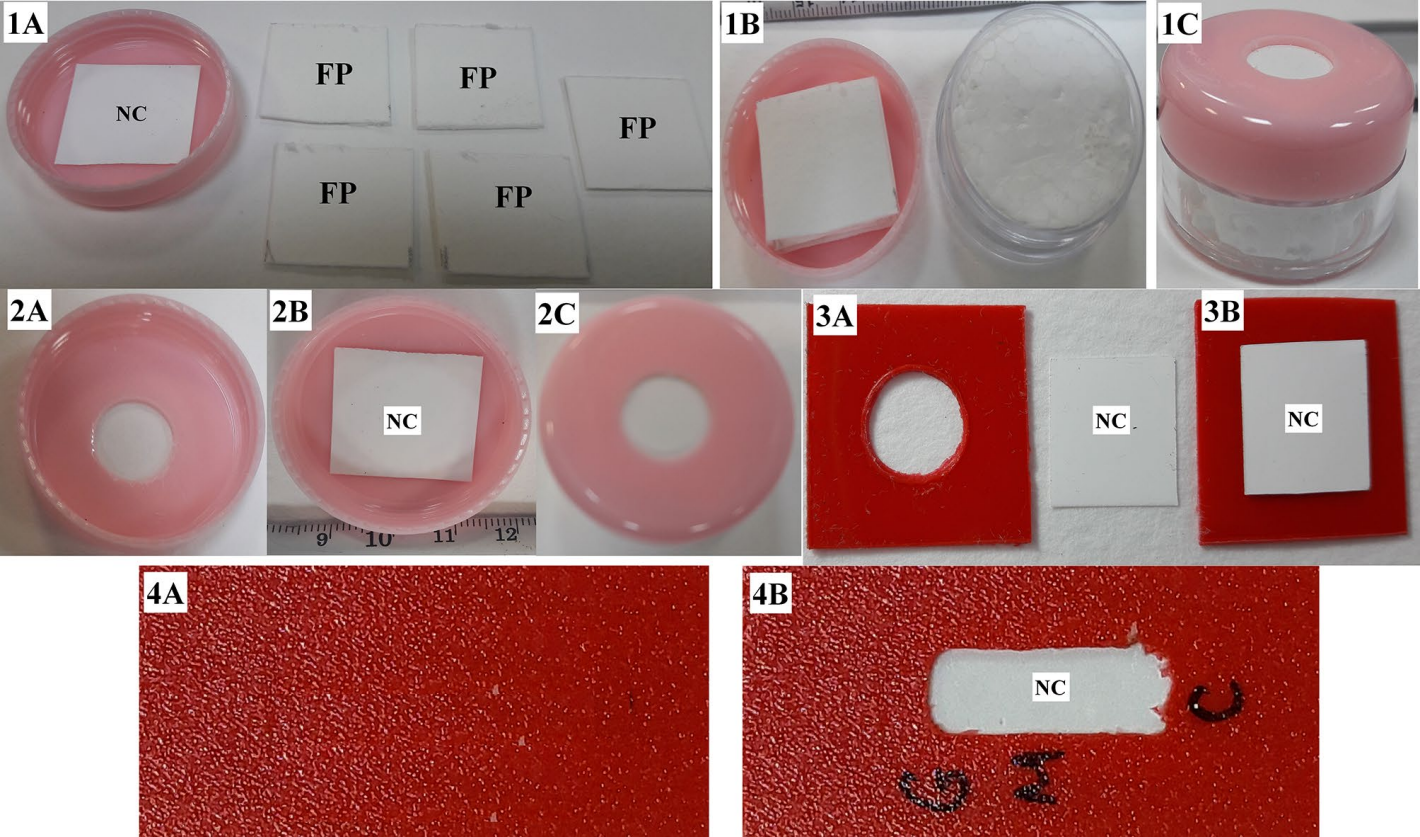
Components of TLTD. Format 1 (1A-1C); Format 2 (2A-2C); Format 3 (3A-3B) and Format 4 (4A-4B). Where NC: Nitrocellulose membrane covering the plastic opening; FP: absorbing filter papers. Format 1 is formed of a cap with a circular opening, a nitrocellulose membrane put into the inside of this cap to cover its opening (1A), FP put over the NC (1B), and the cap containing the NC and FP are put on the container body to get the final shape of the TLTD format 1 (1C). Format 2 is formed of only a cap (2A) and a NC (2B) affixed with a tape into the inside of the cap to cover its opening (2C). Format 3 is formed of only a square piece of plastic with circular hole in its center (3A) and a NC affixed with a tape to into one side to cover its opening (3B). Format 4 is formed of only a rectangular piece of plastic (4A) with a centered rectangular opening and a NC affixed with a tape at one side to cover its opening (4B).

Format 1: A container with a screw cap was used to assemble the TLTD (Fig. 6). The cap has a ring opening of 12 mm diameter at the center. The container was filled with several layers of square absorbing filter papers (Thomas Scientific). A nitrocellulose (NC) piece (0.45 µm, GE Healthcare—Life Sciences) is laid on the top of the absorbing filter papers and the cap is closed to hold the NC paper firmly in contact with the absorbing filter papers.

Format 2: only the cap of format 1 was used but the NC paper was sealed with an adhesive tape to block the hole from the inside of the cap.

Format 3: A square piece of flat plastic with a hole in its center was used. One side of the hole is blocked using a piece of NC membrane and adhesive tape.

Format 4: The same design as that of format 3 but with rectangular opening.

### Principle and mechanism of TLTD workings

Nitrocellulose paper is manually blotted with 1 µL of goat anti-human IgG Fc Abs (1 mg/mL, R&D Systems), 1 µL of goat anti-human IgM Abs (1 mg/mL, Vector Laboratories, Inc.) and 1 µL of rabbit serum (Sigma-Aldrich) as a positive control and left to dry at 37 $^\circ$ C for 1 h. The NC was blocked with blocking buffer (PBS containing 2% BSA, 0.5% Teween-20) for 3 h. The NC was washed with washing buffer (PBS containing 0.5% Teween-20) and left to dry. The NC was then assembled into the device as described above.

**Figure 7:**
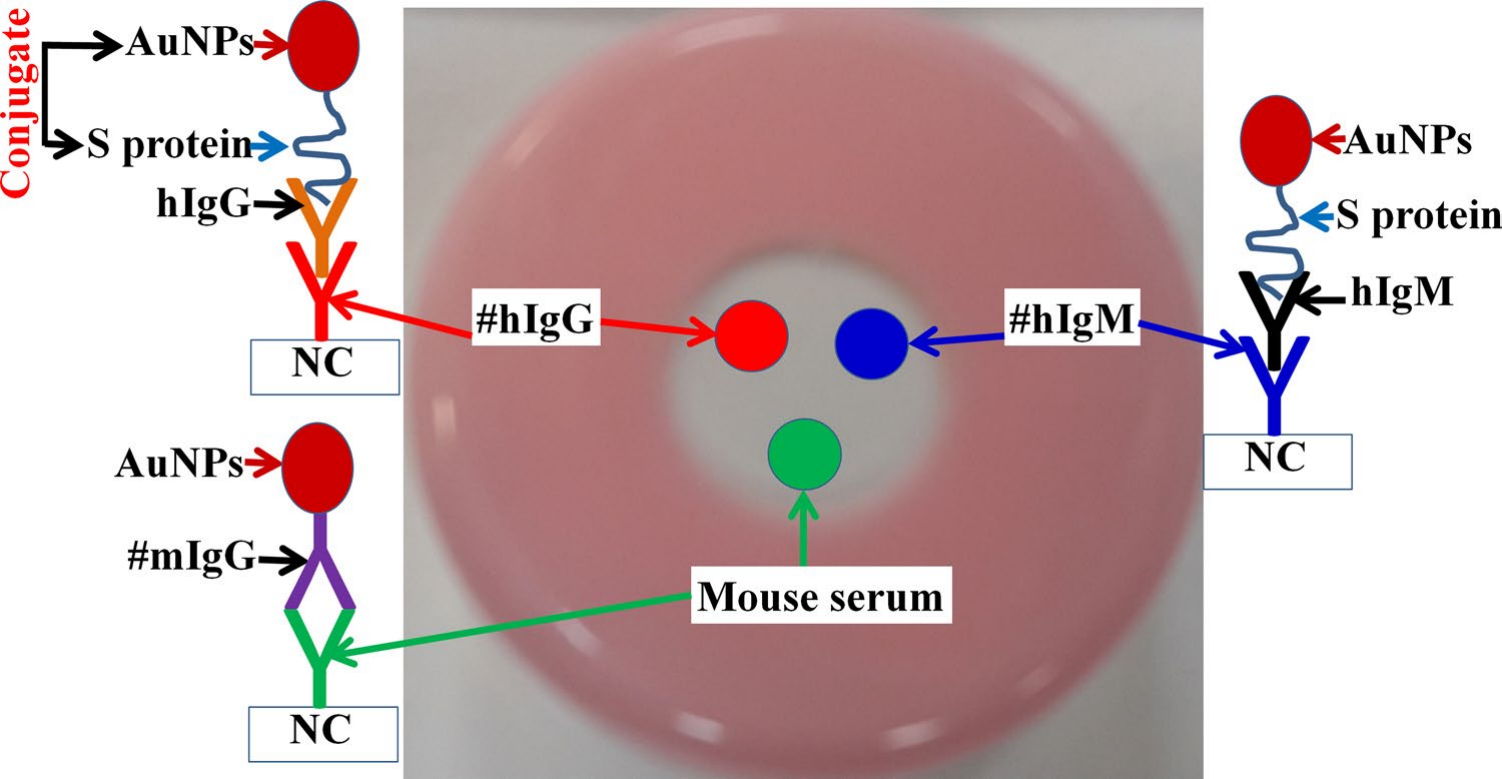
Principle structure and work of the TLTD. Where NC: nitrocellulose membrane; #hIgG: anti-human IgG Abs; hIgG: human IgG; #hIgM: anti-human IgM Abs; hIgM: human IgM; #rIgG: anti-rabbit IgG Abs. AuNPs: Gold nanoparticles.

The principle of the detection of anti-SARS-CoV-2 IgG and IgM Abs using all formats is shown in Fig. 7.

Format 1: Human serum is being added to the hole of the TLTD directly onto NC membrane and left to pass through by the action of gravity and the absorbing filter papers below the NC. IgG and IgM which are present in the human serum will be captured by the corresponding blotted antibodies. The prepared probe (S-AuNPs) mixed with the commercially available goat anti-rabbit IgG − gold antibody (Sigma-Aldrich) are added to the hole of the TLTD directly to NC membrane and left to pass through by the action of gravity and absorbing filter papers below the NC. Approximately 2–3 drops of wash buffer are added to the hole of the TLTD if needed. If any of the captured human IgG or IgM are reactive toward the S protein of SARS-CoV-2, the S-Gold probe will be captured by these antibodies and indicated by the characteristic reddish-brown color of the gold NPs. Anti-rabbit IgG-gold will react with IgG present in the blotted rabbit serum giving a reddish-brown dot (Supplementary video 1).

Regarding formats 2–4, the serum is applied to the hole of the TLTD, left for 1–2 min, liquid is absorbed using absorbing filter paper, mixed gold conjugates are applied to the hole of the TLTD, left for 1–2 min, liquid is absorbed using absorbing filter paper and washed twice by adding wash buffer to the hole and absorbing the buffer with absorbing filter paper (Supplementary video 2).

### Samples

The Research Ethics Committee at King Khalid University (HAPO-06-B-001) reviewed and agreed on this study. All experiments were performed in accordance with the above mentioned committee guidelines and regulations. Patient informed consent was not a need as we got coded serum samples without patient information.

We used patient sera that were diagnosed positive for COVID-19 via RT-PCR (n = 89). We have stored coded sera (n = 7267 random healthy blood donor volunteers) for several years before the onset of the pandemic and were used in a previous study (https://journals.ekb.eg/article_13699.html). From these samples, negative control sera (n = 100, HIV, HCV, HBV, HTLV and cytomegalovirus-free), HCV positive (antibody and PCR confirmed, n = 5), HBV positive (surface antigen and PCR confirmed, n = 50) and cytomegalovirus positive (antibody confirmed, n = 20) were selected.

All samples used in this work were tested for anti-SARS-CoV-2 IgG and IgM Abs using COVID-19 Human IgM IgG Assay Kit (Abnova). The ELISA plates of the Abnova kit are coated with the S protein, similar to the S-Gold probe used in this work. All samples were also tested for the presence of anti-SARS-CoV-2 IgG and IgM Abs using the four TLTD formats as described above.

### Sensitivity of TLTD

For titration purpose, sera were serially diluted in wash buffer (1:2, 1:4, 1:8, 1:16, 1:32, 1:64 and 1:128). The outcomes were decided as positive or negative by visual review and the test detection limit was identified as the minimum concentration of the serum at which the test dot was unambiguous.

### Specificity of TLTD

To determine the specificity of the TLTD for COVID-19 detection, normal and HCV, HBV, HIV, and cytomegalovirus-infected sera were tested using TLTD.

### TLTD stability and reproducibility

COVID-19 (positive and negative) confirmed samples were examined 3 times to explore the reproducibility of TLTD.

For stability, TLTDs were kept at 4 °C, ambient temperature and 37 °C (accelerated heat stability) up to 30 days and examined using positive/negative samples at the end of incubations.

## Supplementary Information

Supplementary Information 1.

Supplementary Information 2.

Supplementary Information 3.

Supplementary Information 4.

Supplementary Information 5.

Supplementary Information 6.

Supplementary Video 1.

Supplementary Video 2.

## Data Availability

All data are available in the manuscript and supplementary files.

## References

[1] Riera Romo, M., Pérez-Martínez, D. & Castillo Ferrer, C. Innate immunity in vertebrates: An overview. *Immunology* **148**, 125–139 (2016). 10.1111/imm.12597 PMC4863567 26878338

[2] Alvarez JI (2005). Inhibition of toll like receptor immune responses by microbial pathogens. Front. Biosci..

[3] Iwasaki A, Medzhitov R (2004). Toll-like receptor control of the adaptive immune responses. Nat. Immunol..

[4] Elgueta R, De Vries VC, Noelle RJ (2010). The immortality of humoral immunity. Immunol. Rev..

[5] Kumar BV, Connors TJ, Farber DL (2018). Human T Cell Development, Localization, and Function throughout Life. Immunity.

[6] Wang C, Horby PW, Hayden FG, Gao GF (2020). A novel coronavirus outbreak of global health concern. The Lancet.

[7] Huang, Y., Yang, C., Xu, X. feng, Xu, W. & Liu, S. wen. Structural and functional properties of SARS-CoV-2 spike protein: potential antivirus drug development for COVID-19. *Acta Pharmacol. Sin.* **41**, 1141–1149 (2020). 10.1038/s41401-020-0485-4 PMC7396720 32747721

[8] Tan W (2020). A novel coronavirus genome identified in a cluster of Pneumonia Cases — Wuhan, China 2019–2020. China CDC Wkly..

[9] Walls AC (2020). Structure, function, and antigenicity of the SARS-CoV-2 spike glycoprotein. Cell.

[10] Tang NLS (2005). Early enhanced expression of interferon-inducible protein-10 (CXCL-10) and other chemokines predicts adverse outcome in severe acute respiratory syndrome. Clin. Chem..

[11] Ma H (2020). Serum IgA, IgM, and IgG responses in COVID-19. Cell. Mol. Immunol..

[12] Zhao J (2020). Antibody responses to SARS-CoV-2 in patients with novel coronavirus disease 2019. Clin. Infect. Dis..

[13] Grifoni A (2020). Targets of T cell responses to SARS-CoV-2 coronavirus in humans with COVID-19 disease and unexposed individuals. Cell.

[14] Weiskopf D (2020). Phenotype and kinetics of SARS-CoV-2-specific T cells in COVID-19 patients with acute respiratory distress syndrome. Sci. Immunol..

[15] Wang F (2020). Characteristics of peripheral lymphocyte subset alteration in COVID-19 pneumonia. J. Infect. Dis..

[16] Chen G (2020). Clinical and immunological features of severe and moderate coronavirus disease 2019. J. Clin. Invest..

[17] Wu F (2020). A new coronavirus associated with human respiratory disease in China. Nature.

[18] Ni L (2020). Detection of SARS-CoV-2-specific humoral and cellular immunity in COVID-19 convalescent individuals. Immunity.

[19] Wang X (2020). Neutralizing antibody responses to severe acute respiratory syndrome coronavirus 2 in coronavirus disease 2019 inpatients and convalescent patients. Clin. Infect. Dis..

[20] Wu F (2020). Neutralizing antibody responses to SARS-CoV-2 in a COVID-19 recovered patient cohort and their implications. SSRN Electron. J..

[21] Amanat F (2020). A serological assay to detect SARS-CoV-2 seroconversion in humans. Nat. Med..

[22] Ng KW (2020). Preexisting and de novo humoral immunity to SARS-CoV-2 in humans. Science.

[23] Sethuraman N, Jeremiah SS, Ryo A (2020). Interpreting Diagnostic Tests for SARS-CoV-2. JAMA J. Am. Med. Assoc..

[24] Quesada-González D, Merkoçi A (2015). Nanoparticle-based lateral flow biosensors. Biosens. Bioelectron..

[25] Oishee MJ (2021). Covid-19 pandemic: Review of contemporary and forthcoming detection tools. Infect. Drug Resist..

[26] Sajid M, Kawde AN, Daud M (2015). Designs, formats and applications of lateral flow assay: A literature review. J. Saudi Chem. Soc..

[27] Temperton NJ (2005). Longitudinally profiling neutralizing antibody response to SARS coronavirus with pseudotypes. Emerg. Infect. Dis..

[28] Zhang L (2006). Antibody responses against SARS coronavirus are correlated with disease outcome of infected individuals. J. Med. Virol..

[29] Cao Z (2010). Potent and persistent antibody responses against the receptor-binding domain of SARS-CoV spike protein in recovered patients. Virol. J..

[30] Ballarin B (2010). Gold nanoparticle-containing membranes from in situ reduction of a gold(III)-aminoethylimidazolium aurate salt. J. Phys. Chem. C.

[31] Sonavane G, Tomoda K, Makino K (2008). Biodistribution of colloidal gold nanoparticles after intravenous administration: Effect of particle size. Colloids Surfaces B Biointerfaces.

[32] Long QX (2020). Antibody responses to SARS-CoV-2 in patients with COVID-19. Nat. Med..

[33] Oliveira BA, de Oliveira LC, Sabino EC, Okay TS (2020). SARS-CoV-2 and the COVID-19 disease: A mini review on diagnostic methods. Rev. Inst. Med. Trop. Sao Paulo.

[34] Luppa PB, Müller C, Schlichtiger A, Schlebusch H (2011). Point-of-care testing (POCT): Current techniques and future perspectives. TrAC - Trends Anal. Chem..

[35] Yu X (2018). Development of colloidal gold-based immunochromatographic assay for rapid detection of goose parvovirus. Front. Microbiol..

[36] Goarant C (2013). Sensitivity and Specificity of a New Vertical Flow Rapid Diagnostic Test for the Serodiagnosis of Human Leptospirosis. PLoS Negl. Trop. Dis..

[37] Ross GMS, Salentijn GI, Nielen MWF (2019). A critical comparison between flow-through and lateral flow immunoassay formats for visual and smartphone-based multiplex allergen detection. Biosensors.

[38] Gao Z (2016). Development of a new limiting-antigen avidity dot immuno-gold filtration assay for HIV-1 incidence. PLoS ONE.

[39] Frens G (1973). Controlled Nucleation for the Regulation of the Particle Size in Monodisperse Gold Suspensions. Nat. Phys. Sci..

[40] Thian ES (2013). Zinc-substituted hydroxyapatite: A biomaterial with enhanced bioactivity and antibacterial properties. J. Mater. Sci. Mater. Med..

