# Supplementary material for: Development of rapid and cost-effective top-loading device for the detection of anti-SARS-CoV-2 IgG/IgM antibodies
Source: Sci Rep. 2021 Jul 21;11:14926. doi: 10.1038/s41598-021-94444-6 (PMC8295295; doi:10.1038/s41598-021-94444-6)
Supplement: Supplementary file 1 — Supplementary Information 1. [file 41598_2021_94444_MOESM1_ESM.docx]

**Development of rapid and cost-effective top-loading device for the detection of anti-SARS-CoV-2 IgG/IgM antibodies**

**Essam H. Ibrahim^1,2,3*^, Hamed A. Ghramh^1,2,4^, Mona Kilany^2,5^**

^1^ Biology Department, Faculty of Science, King Khalid University, P.O. Box 9004, Abha 61413, Saudi Arabia.

^2^ Research Center for Advanced Materials Science (RCAMS), King Khalid University, P.O. Box 9004, Abha 61413, Saudi Arabia.

^3^ Blood Products Quality Control and Research Department, National Organization for Research and Control of Biologicals, Cairo, Egypt.

^4^ Unit of Bee Research and Honey Production, Faculty of Science, King Khalid University, P.O. Box 9004, Abha 61413, Saudi Arabia.

^5^ Department of Microbiology, National Organization for Drug Control and Research (NODCAR), Cairo, Egypt.

**Supplementary** **Figures**


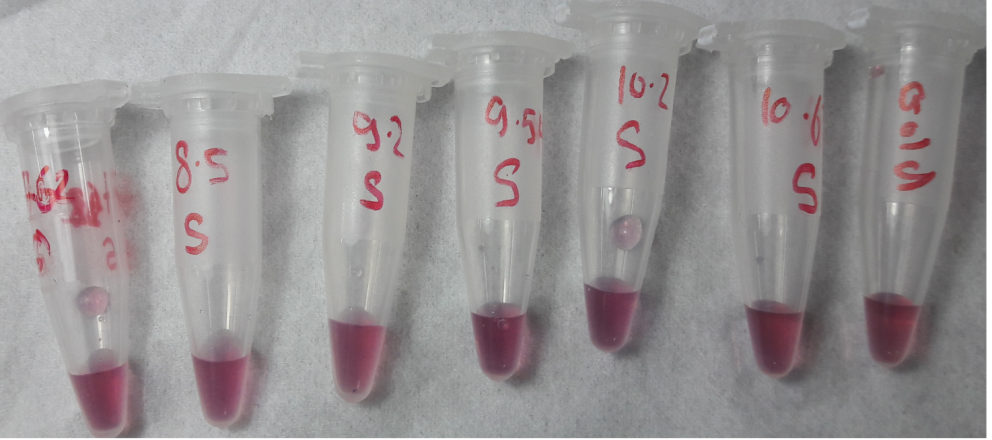


**Supplementary** **Fig. S1:** Testing of optimal pH for S protein conjugation with AuNPs. Where: S: S protein; numbers: indicate pH value at which conjugation tested and gold: untreated AuNPs.


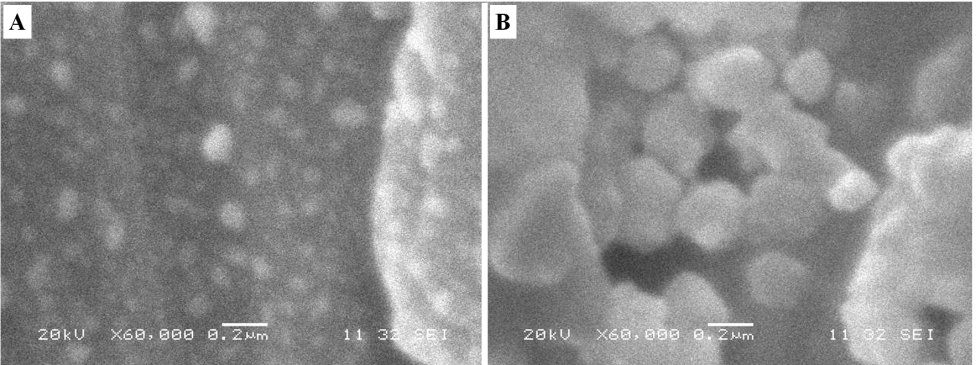


**Supplementary** **Fig. S2**: SEM image showing the spherical AuNPs. A: before conjugation and B: after conjugation with S protein.


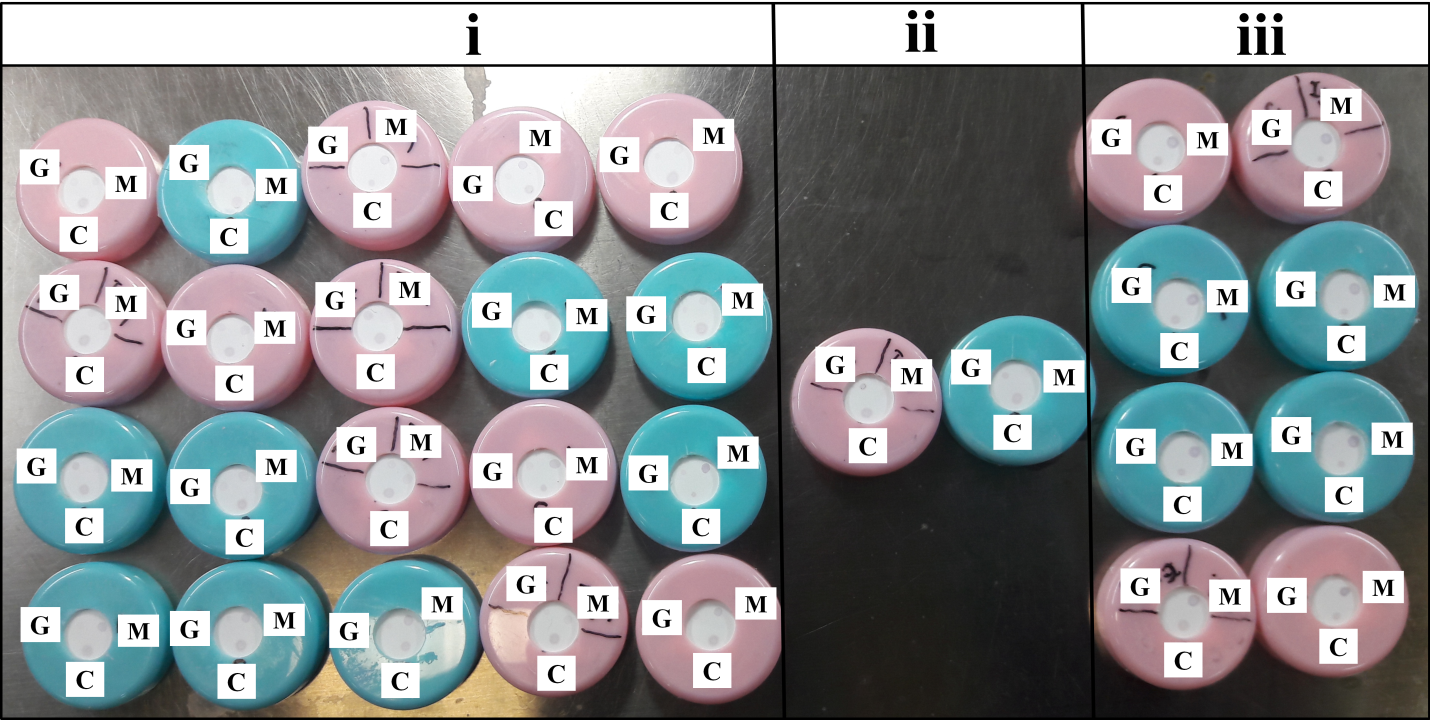


**Supplementary** **Fig. 3**: Testing of COVID-19 RT-PCR positive sera using TLTD. Column i: Samples positive for both anti-SARS-CoV-2 IgG and IgM Abs; Column ii: Samples positive for anti-SARS-CoV-2 IgG Abs only and Column iii: Samples positive for anti-SARS-CoV-2 IgM Abs only. Where: G: expected positive area for IgG against S protein; M: expected positive area for IgM against S protein and C: positive control. All sera at the dilution 1:4.

**
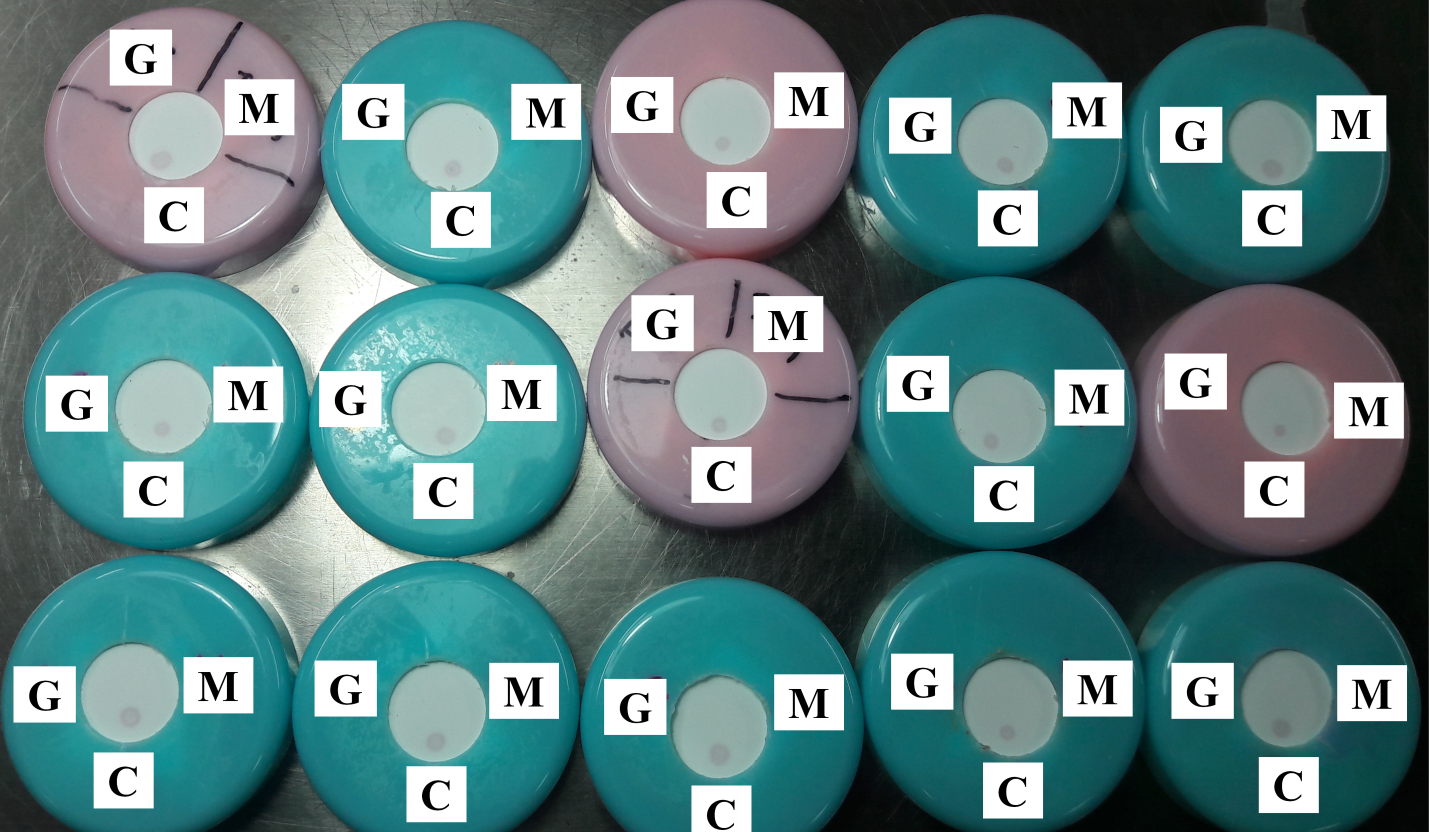
**

**Supplementary** **Fig. S4**: Testing of HCV, HBV, HIV and cytomegalovirus-infected sera using TLTD. Where G: expected positive area for IgG against S protein; M: expected positive area for IgM against S protein and C: positive control. All sera at the dilution 1:4.


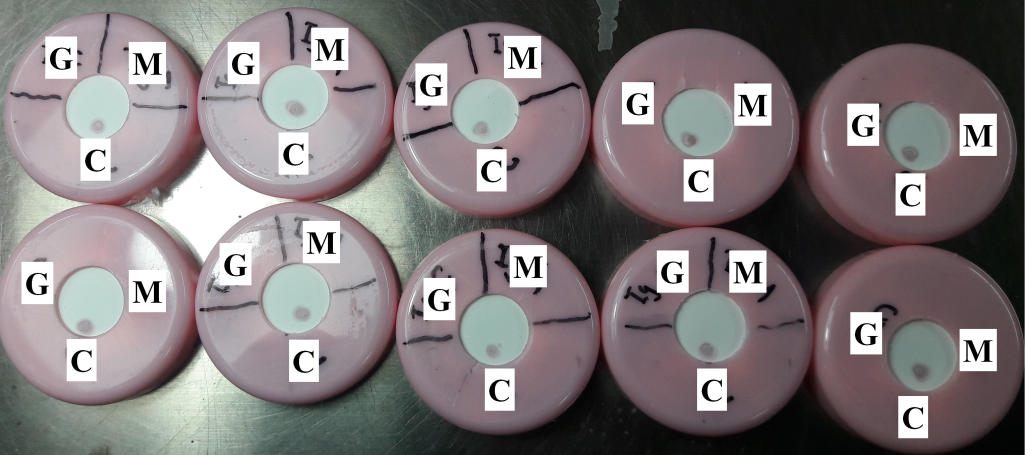


**Supplementary** **Fig. S5**: Testing of Syphilis, malaria, HCV, HBV, HIV, HTLV and cytomegalovirus-free sera using TLTD. Where G: expected positive area for IgG against S protein; M: expected positive area for IgM against S protein and C: positive control. All sera at the dilution 1:4.

.
