# Supplementary figures and images for: Development of rapid and cost-effective top-loading device for the detection of anti-SARS-CoV-2 IgG/IgM antibodies
Source: Sci Rep. 2021 Jul 21;11:14926. doi: 10.1038/s41598-021-94444-6 (PMC8295295; doi:10.1038/s41598-021-94444-6)

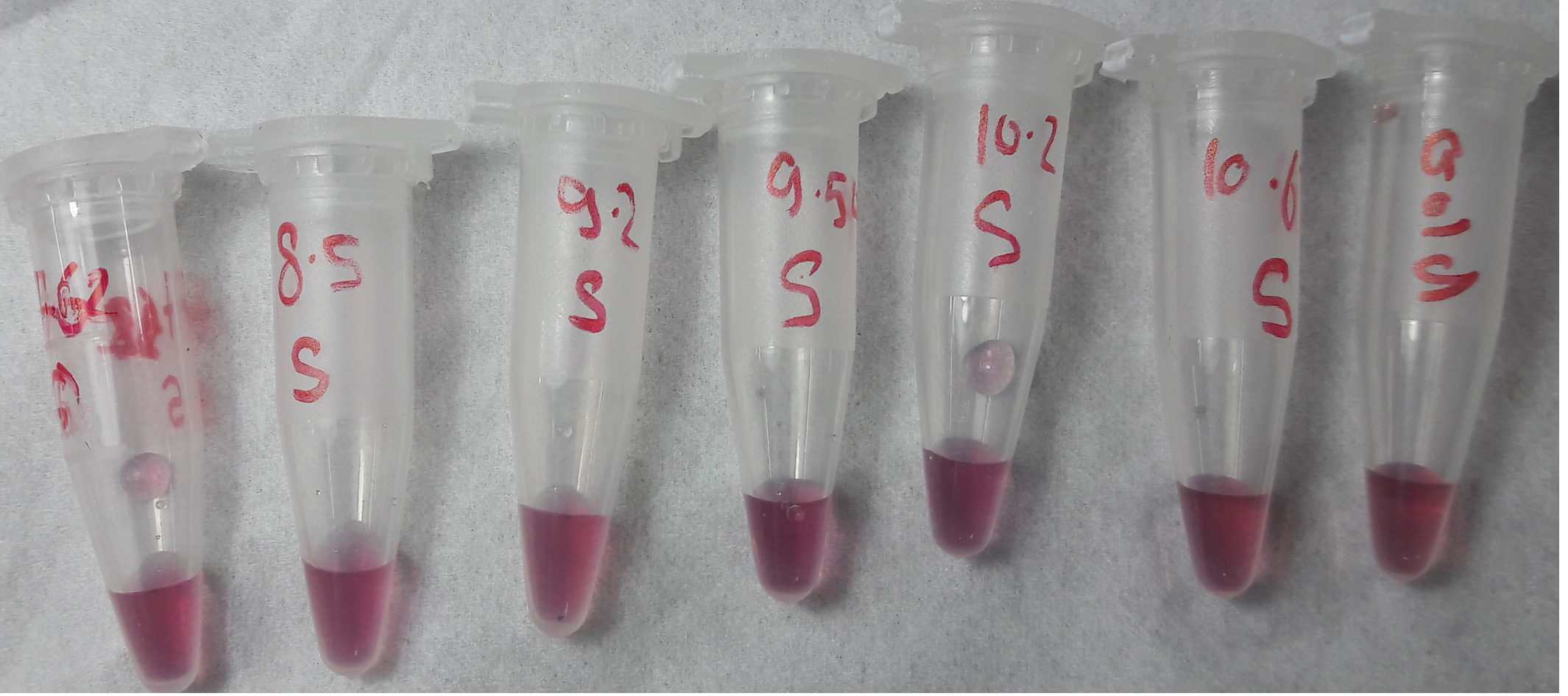

Supplement: Supplementary file 2 — Supplementary Information 2. [file 41598_2021_94444_MOESM2_ESM.tif]

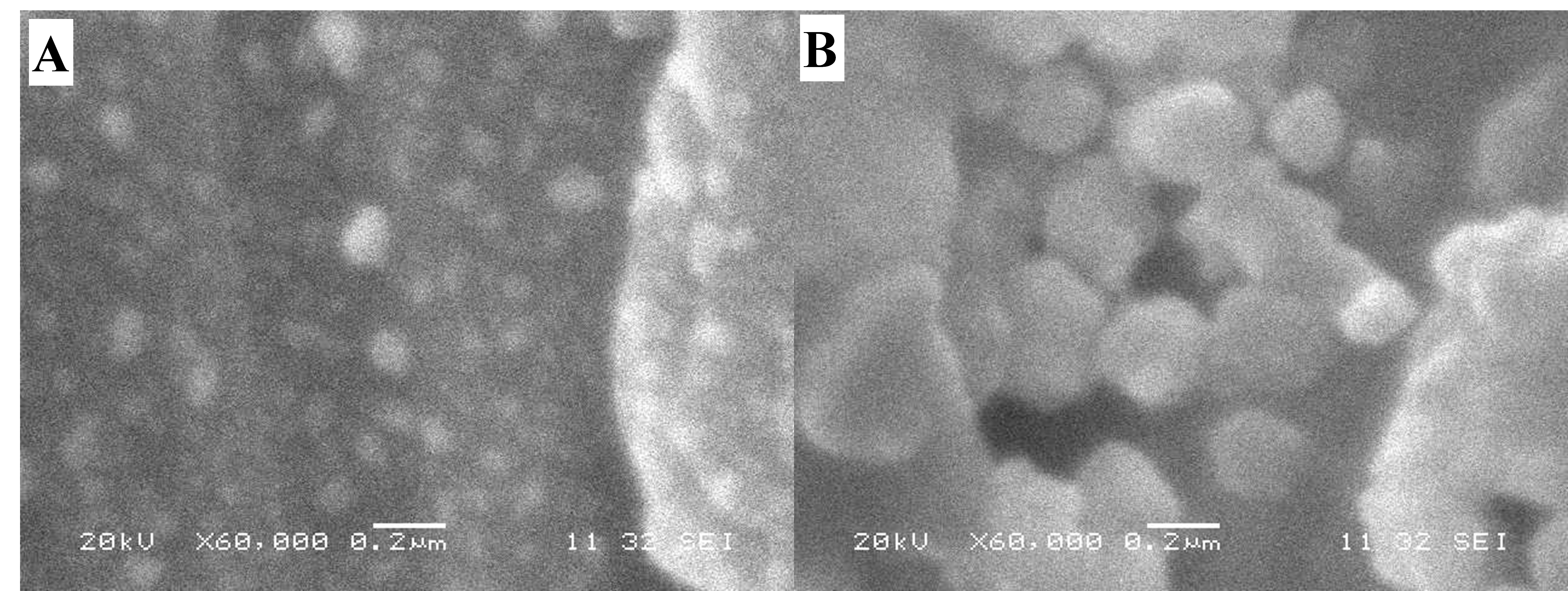

Supplement: Supplementary file 3 — Supplementary Information 3. [file 41598_2021_94444_MOESM3_ESM.tif]

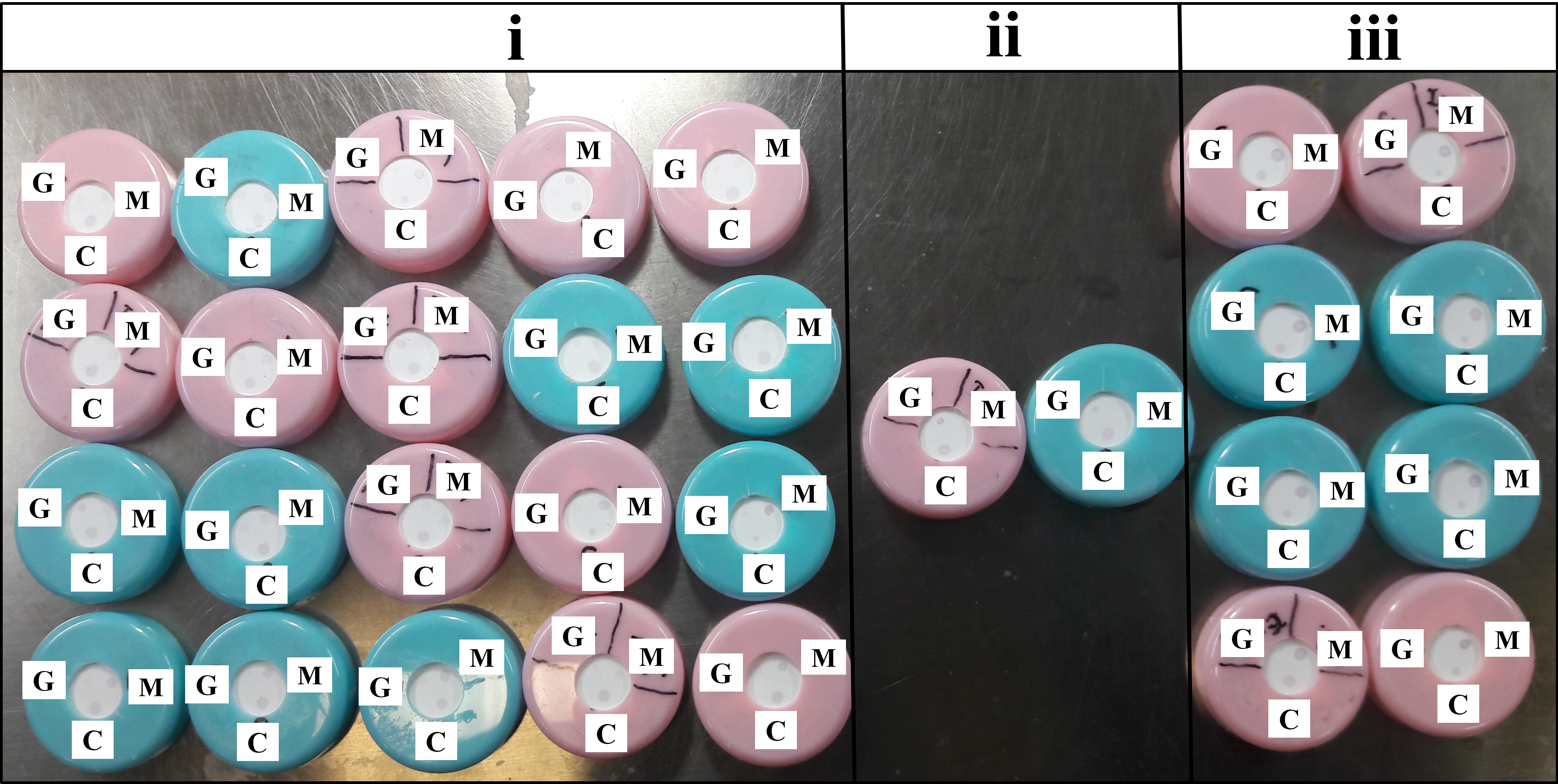

Supplement: Supplementary file 4 — Supplementary Information 4. [file 41598_2021_94444_MOESM4_ESM.tif]

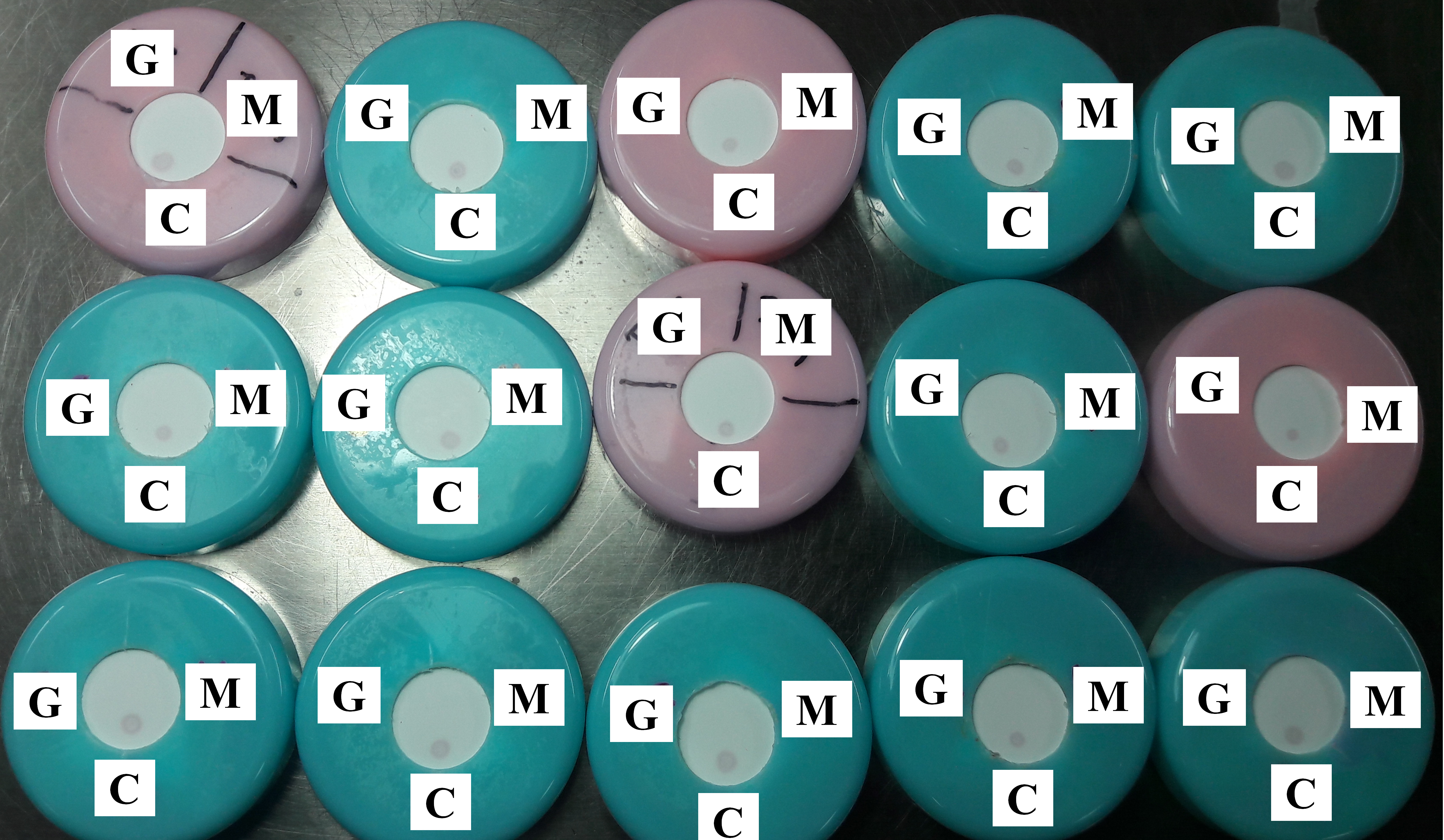

Supplement: Supplementary file 5 — Supplementary Information 5. [file 41598_2021_94444_MOESM5_ESM.tif]

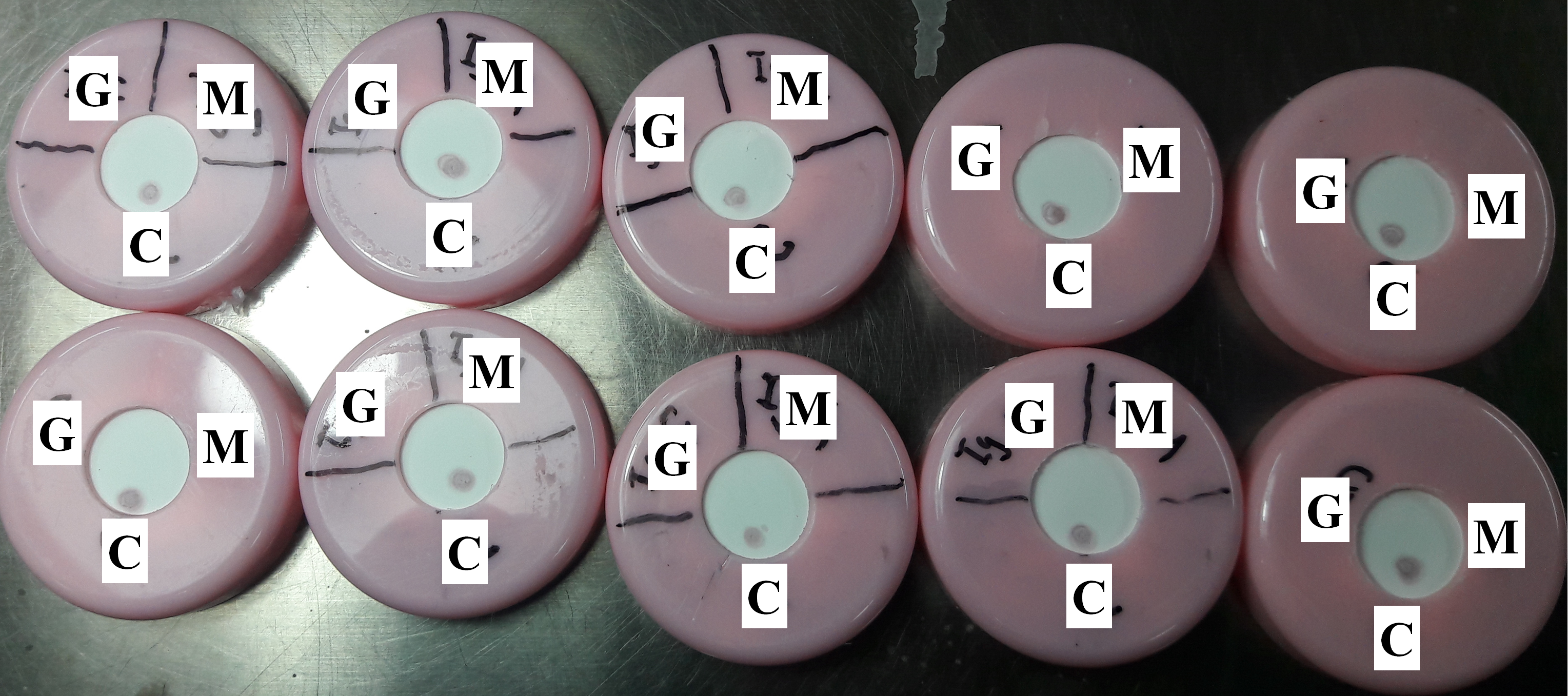

Supplement: Supplementary file 6 — Supplementary Information 6. [file 41598_2021_94444_MOESM6_ESM.tif]
